# Association of Diet, Body Mass Index, and Lifestyle on the Gastrointestinal Health Risk in a Sample of Adults

**DOI:** 10.3390/ijerph191710569

**Published:** 2022-08-25

**Authors:** Reyna Sámano, Fernanda Esparza-Juárez, Gabriela Chico-Barba, Erika González-Medina, Bernarda Sánchez-Jiménez, María Hernández-Trejo

**Affiliations:** 1 Coordinacion de Nutricion y Bioprogramacion, Instituto Nacional de Perinatologia, Secretaria de Salud, Mexico City 11000, Mexico; 2 Programa de Posgrado Doctorado en Ciencias Biologicas y de la Salud, Division de Ciencias Biologicas y de la Salud, Universidad Autonoma Metropolitana, Mexico City 04960, Mexico; 3 Escuela de Dietetica y Nutricion del ISSSTE, Mexico City 14070, Mexico; 4 Escuela de Enfermeria, Facultad de Ciencias de la Salud, Universidad Panamericana, Mexico City 03920, Mexico; 5 Departamento de Neurobiología del Desarrollo, Instituto Nacional de Perinatologia, Secretaria de Salud, Mexico City 11000, Mexico

**Keywords:** eating habits, obesity, exercise, intestinal symptoms, World Gastroenterology Organization Test

## Abstract

Gastrointestinal functional disorders are characterized by abnormalities in motility with visceral hypersensitivity, representing a global public health problem. We aimed to determine whether eating habits, lifestyle characteristics, and body mass index (BMI) are associated with gastrointestinal health risk. The Gastrointestinal Health (GIH) test of the World Gastroenterology Organization (WGO) and the Roma IV criteria were applied. We obtained information on food consumption habits and aerobic exercise, among other variables. Not exercising regularly, drinking water and eating vegetables less than recommended, having high body weight, and taking symptomatic medication were variables that explained 73% of the probabilities of not having good GIH (R^2^ = 0.734). According to Rome IV criteria, women had a 50% higher risk than men of having functional bowel disorder (RR 1.6, 95% CI: 1.04, 2.45). Among the men studied, eating few or no vegetables and drinking less than 1 L of water daily was more frequent; however, the women had significantly more intestinal symptoms. In addition, constipation was higher among women than men (*p* = 0.020). All of the above explains the prognostic value of eating habits and the importance of paying attention to body weight to reduce the risk of gastrointestinal disease.

## 1. Introduction

The gastrointestinal function includes motility, digestion, secretion, perfusion, the modulation of the absorption barrier, and many endocrinological and immune activities under intense interaction with the intestinal microbiota. The intestinal system has great plasticity, and its dysregulation contributes to developing of multiple pathologies of gastrointestinal function, the immune and cardiovascular systems, and metabolic and degenerative alterations [1].

Functional gastrointestinal disorders (FGID) are common in the general population. A large part of the Western population suffers from intestinal disorders, commonly referred to as functional [2]. Gastrointestinal functional alterations are common and include the so-called functional dyspepsia and irritable bowel syndrome (IBS), which affect up to 28% of the global population, being more frequent in advanced age [3,4]. According to the World Gastroenterology Organization (WGO), gastrointestinal symptoms are recurrent or chronic symptoms of any digestive system organ, yet little is known about the individual epidemiology of each symptom. Since 1993, the trend has been to develop criteria to categorize symptoms as functional digestive disorders or FGID. Some of these are related to overweight and obesity through mechanical factors, such as increased abdominal pressure. There are also systemic factors since visceral fat releases pro-tumoral and pro-inflammatory cytokines such as tumor necrosis factor and interleukins 1 and 6. People with overweight and obesity present the inhibition of small bowel motility and delay intestinal gas transit. Additionally, adipocytes-released peptides promote GI motor disorders [5,6].

In addition, multiple studies suggest that eating habits and diet may be related to the pathophysiology of functional intestinal disorders and functional dyspepsia [7,8,9]. Nevertheless, little is known because little research has been done in our region on associations of intestinal discomfort with the usual diet of the population [10,11]. Therefore, this study analyzed the frequency of intestinal symptoms, individually and as functional digestive disorders, in adults. In addition, we aimed to determine whether eating habits, some lifestyle characteristics, and body mass index (BMI) may be associated with the gastrointestinal health categories [5,12].

## 2. Materials and Methods

### 2.1. Study Population

We performed a cross-sectional study. We included 250 participants, they were selected consecutively and not randomly from the patients who attended a Nutrition and Dietetics Clinic consultation for social security for state workers beneficiaries located in Mexico City. Inclusion criteria were workers between 22 and 65 years; without hypertensive disease, diabetes, or another chronic degenerative disease; functional bowel disease; and who freely signed the informed consent form. The research was carried out under the principles of the Declaration of Helsinki, maintaining the confidentiality of the study subjects at all times. Furthermore, according to the Mexican General Health Law on Health Research [13], the investigation was considered to be without risk and had a registered number by Institutional Committees (212250-08361).

### 2.2. Sociodemographic, Habits, and Lifestyle Data

A survey was designed for this study to obtain the following information: (1) demographic and socioeconomic factors; (2) water consumption habits and certain groups of foods, natural and industrialized, as well as soft drinks, alcoholic beverages, and coffee. In addition, the places, times, and the number of meals made per day were investigated; (3) lifestyle, such as aerobic exercise, smoking, and self-perceived stress; and (4) the use of symptomatic gastro-enteric medications.

### 2.3. Gastrointestinal Health

A gastrointestinal health (GIH) test designed by the WGO [3,12] was applied. The test consists of 8 Likert-type questions on habits related to digestive health. The GIH test classifies as Good Digestive Health when the total score is greater than 15, and a score between 5 and 15 is considered a high risk of affecting digestive health. A score below five is considered Poor Digestive Health and needs prompt medical attention. https://www.worldgastroenterology.org/forms/health-test.php (accessed on 19 August 2022).

### 2.4. Functional Gastrointestinal Disorders

The Rome IV criteria were used to detect Irritable Bowel Syndrome (IBS) [14]. IBS was defined when participants met the following criteria: Recurrent abdominal pain at least one day a week in the last three months, associated with one or more of the following symptoms: 1. being related to defecation; 2. being associated with a change in stool frequency; and 3. being associated with a change in the shape or appearance of the stool. These criteria must be met during the last three months, with the appearance of symptoms at least six months before diagnosis [12]. To classify cases of IBS with intestinal constipation, our team used the criteria applied by Mearin and Cols. [15].

### 2.5. Body Mass Index (BMI)

Participants’ anthropometric measurements were obtained by trained nutritionists using the Lohman technique [16]. Under standard conditions body weight was obtained with a digital scale (TANITA, Tokyo, Japan, model BWB-800, accuracy 0.10 kg). Height was assessed using a stadiometer (SECA, Hamburg, Germany, model 208, accuracy 0.1 cm). Then, BMI was calculated by dividing weight in kilograms by the height in square meters and was categorized according to the World Health Organization cutoff points underweight < 18.5, normal weight 18.5–24.99, overweight 25–29.99, obese ≥ 30 [17].

### 2.6. Statistical Analysis

Descriptive statistics, frequencies, and percentages were carried out. We calculated means with standard deviation for continuous and medians and interquartile ranges for other numerical variables with non-normal distribution. The prevalence ratio as a risk ratio and the Chi-square test was calculated to compare groups according to the categories of the GIH test. Additionally, correlations between GIH test score and weight, body mass index, and age were assessed using Pearson’s correlation coefficient.

We performed a univariate general linear model, including logistic regression analysis (LRA), using categorical covariates with a simple contrast method to recognize the magnitude of the participants’ habits. We included 4 of the GI health habits listed in the WGO test. The GIH test score or rating was used as a dependent (outcome) variable. Additionally, it enabled using the results of the GIH test as a dependent variable for relative risk tests.

## 3. Results

Two hundred fifty patients met the inclusion criteria and agreed to participate in the study. In the study, 51 participants were men and 199 women, and their general clinical and sociodemographic characteristics are shown in Table 1.

We observed that being an employee, married, or 41 years old or more had 30% more possibilities of having a poor intestinal health score (data not shown).

### 3.1. Frequency of Intestinal Symptoms

The frequency of isolated intestinal symptoms showed that abdominal distention and flatulence were the most frequent. Moreover, constipation was more common in women than men (Table 2). In addition, the number of episodes of constipation per month was twice in women than men: 3.22 (EE = 0.35) vs. 1.24 (EE = 0.32), *p* = 0.005.

### 3.2. Differences between Sexes

We observed that age was not statistically different between women and men (37.5 vs. 36.0 years, *p* = 0.43). However, men’s body weight was significantly higher (+8.95 kg, *p* < 0.001). Nevertheless, men and women had a similar BMI (27.06 vs. 27.51 Kg/m^2^ (*p* = 0.58).

The GIH test showed that a similar proportion of women (61.3%) and men (72.5%) had a high risk of affecting their digestive health (RR = 0.8, 95% CI 0.69, 1.03, *p* = 0.13). We found that the zero or very low fruits and vegetable intake were 21.7% more prevalent in men than women. Furthermore, men showed 2.6 more probability of avoiding the fruits and vegetables in their daily diet than women (95% CI: 1.31, 5.20).

On the other hand, 100 women (50.3%) and 16 men (31.4%) met the Rome IV criteria for the diagnosis of irritable bowel syndrome or functional bowel disorder; the women had a 50% higher risk of having this diagnosis than men (RR = 1.60, 95% CI 1.04, 2.45, *p* = 0.01). In addition, we saw significant statistical differences in water intake between gender: 80% of men vs. 60% of the women consumed less than 1 L a day (OR 2.64. 95% CI: 1.25, 5.58).

### 3.3. Risks to Intestinal Health

The GIH test results reported that none of the participants scored less than 5. One hundred and fifty-nine participants (63%) scored between 5 and 15, considered by the WGO to have a high risk to digestive health, and 91 participants (36%) had a score greater than 15, which means having good digestive health.

The mean body weight of the sample population was 71.68 kg (SD ± 14.2), and the mean GIH test score was 14.3 (SD ± 3). The correlation between both was moderate, inverse, and significant (r = −0.365, *p* < 0.001). Likewise, the body mass index and the GIH test score correlated (r = −0.432, *p* < 0.001).

Table 3 shows the reduction in the GIH score of intestinal health by BMI and other associated risky eating behaviors. The low or no consumption of vegetables and fruits and the body weight varied inversely to the test score. Physical exercise explained 40% of the model, and the WGO score is reduced to more than 6 points in those who do not practice it. Surprisingly, a paradoxical effect was found among those who use medications to relieve gastrointestinal symptoms: those who took these drugs more frequently had a lower score on their GIH.

Our results highlight that those who had a paid occupation had a 31% higher risk for their intestinal health (RR 1.31, 95% CI 1.06, 1.61). A similar risk (29%) was found in those who lived with a partner (RR 1.29, 95% CI 1.07, 1.57).

Regarding the participants’ BMI, obesity was associated with having greater gastrointestinal symptoms, increasing their probability of high risk of digestive health by progressively lower scores as BMI increases. In contrast, overweight patients did not show differences in their GIH scores compared to those with BMI < 25 kg/m^2^ (Table 3). Furthermore, a proportionally greater risk for their intestinal health was found among those who received medication to reduce gastrointestinal symptoms. Finally, we found that those who did not consume alcoholic beverages at least once a week slightly increased the probability of having a good digestive health rating.

None of the following investigated exposure factors showed statistical significance or association with the desired results (dependent variables): smoking; specific food consumption habits, such as coffee, carbonated drinks, chocolate, breaded foods, and the consumption of irritating foods; and the number of meals per day, place, and time of food consumption.

Furthermore, there was no association of the level of self-perceived stress with the presence of gastrointestinal symptoms and a low GIH score, although we found that the mean stress level in men was 6.0 points, lower than that of women’s mean of 6.9 points (*p* = 0.01).

### 3.4. Rome IV Criteria

Table 4 confirms that the most common symptom condition was intestinal constipation (both in those diagnosed with IBS and those who did not have IBS). Regarding this single symptom (constipation), no associated risk factor was found in the statistical analysis. Additionally, the WGO test score was similar between participants with and without intestinal bowel syndrome, and the WGO test score was similar between participants with and without intestinal constipation.

The protective factors for IBS were alcohol consumption (RR 0.68, 95% CI: 0.48, 0.97) and being male (RR: 0.62, 95% CI: 0.40, 0.95). Conversely, not having the habit of eating dinner increased the risk (RR: 1.48, 95% CI: 1.11, 1.98), and those who consume more than two sodas a week (RR 1.55, 95% CI 1.04, 2.95). The rest of the investigated variables did not show an association with IBS.

**Figure 1:**
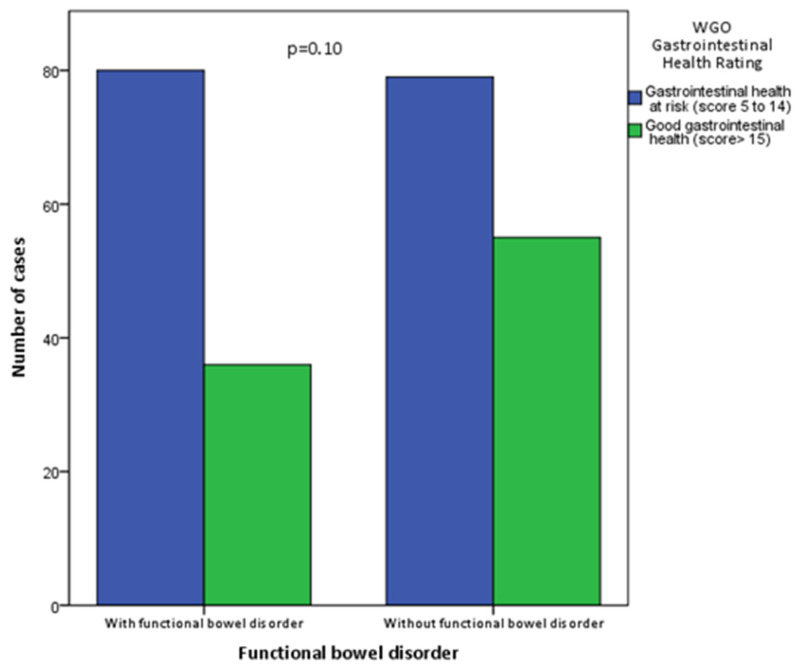
The score of the WGO test in patients with and without functional bowel disorder.

When comparing the results of the GIH test with those of the IBS diagnosis (ROMA IV), it was observed that all patients—with and without criteria for irritable bowel syndrome—had a similar risk for their intestinal health (Figure 1).

It was also interesting to find that the frequency of consumption of gastrointestinal symptomatic medications was very similar between individuals diagnosed with IBS and individuals without this diagnosis (*p* = 0.375).

## 4. Discussion

The results presented here were measured with two well-known gastrointestinal health diagnostic schemes GIH test (WGO) and Rome IV (IBS), which—to the best of our knowledge—had not been reported before. In addition, we made comparisons of both outcome variables, finding discrepancies that we believe are interesting and useful.

Gastrointestinal functional disorders are characterized by abnormalities in motility with visceral hypersensitivity [18]. They affect up to 40% of the world’s population, making it a global public health problem [19]. Most of the participants in our study had some intestinal symptoms.

The most frequent isolated symptom was intestinal constipation, as reported in the international literature [20,21,22,23,24,25]. In addition, women had twice the frequency compared to men.

On the other hand, there were no differences in intestinal constipation between the subjects we studied with and without IBS; the same occurred among those who had and did not have a gastrointestinal risk rating in the GIH test.

### 4.1. Functional Bowel Disorder (Irritable Bowel Syndrome)

The risk of having IBS in the studied sample was double in women than in men, a situation that has been previously identified and reported [9,26]. In this sense, the different levels of self-perceived stress among our participants could contribute to understanding this difference between the sexes, similar to what Tollenar found in his study [27] but with tests that measure stress with greater precision.

### 4.2. Role of Diet on Intestinal Disease

In many patients, functional intestinal disturbances are associated with food intake [28]. Additionally, certain foods can activate the local immune system in certain people and damage the gastrointestinal epithelium [29,30]. On the other hand, some food components can activate specific intestinal receptors and induce the release of neurotransmitters, such as prostaglandin E2 (PGE2), serotonin, glutamate, norepinephrine, gamma-aminobutyric acid (GABA), 5-hydroxy-indol acetic acid, and acetylcholine. While the main hormones released are catecholamines, glucagon-like peptide 1, corticotrophin-releasing hormone (CRH), and melatonin [30,31,32].

Furthermore, diet can significantly affect the gut microbiota, including processed foods [33]. Patients who have functional intestinal disorders and people with inflammatory bowel disease show significant clinical improvement when they follow diets low in FODMAP (fermentable oligosaccharides, disaccharides, monosaccharides, and polyols) [32,34] and, even more, with the dietary protocol 5Ad. Dietary protocol 5Ad proposes, mainly, to exclude all foods that contain large amounts of known offensive elements (oligosaccharides, additives, and highly refined foods) and include products low in lactose with adequate levels of calcium, as well as to consume only fruits with equimolar concentrations of fructose and glucose and ensure a high intake of fruit/vegetables [35].

#### 4.2.1. Alcohol

One of the intentions of carrying out this project was to recognize if any food group had an association with intestinal symptoms, individually or as a syndrome. According to our results also found that alcohol consumption showed a borderline protective effect against gastrointestinal symptoms; alcohol consumption reduced the probability of having IBS by 32%. On the other hand, Reding et al. reported that in patients with inflammatory bowel disease, mainly women, who consume alcohol excessively, have exacerbations of their intestinal symptoms, diarrhea, in a higher proportion [36]. Rozich recently carried out an extensive review of the effect of alcohol consumption on gut health. He refers to studies that saw the worsening of the endoscopic findings of ulcerative colitis among those who ingested beer and wine. On the other hand, he reports a study that showed an increased risk of relapse among those who consumed alcoholic beverages, especially those containing sulfites [37]. Additionally, some studies agree that there is little or no association between alcohol consumption and the development of inflammatory bowel disease. Thus, Bergmann et al. conducted extensive research with more than 260,000 participants without finding an association between alcohol use and the risk of developing IBS [38].

In addition, the meta-analysis by Yang et al. also found no significant association between alcohol intake and the risk of Crohn’s disease [39].

Therefore, our findings should be considered with reserve. To gain more knowledge on this aspect, it would be useful to design studies on the effect of alcohol consumption on the intestine.

#### 4.2.2. Fruits and Vegetables

Fruits, legumes, whole grains, and vegetables are sources of regular dietary fiber, the benefits of which on human GIH are well known [40,41]. The concept that low fiber intake is one of the causes of IBS has been widely spread. In clinical practice, it is recommended to increase fiber intake by fruits and vegetables intake [42].

However, there are different types of dietary fiber with different properties and health benefits. Highly fermentable, short-chain soluble dietary fiber, such as oligosaccharides, results in rapid gas production that can cause abdominal pain, bloating, and flatulence in IBS patients. In contrast, moderately fermentable, soluble, intermediate-viscosity, long-chain dietary fiber has low gas production and the absence of related symptoms [43]. That is why in recent years the idea has been introduced that consuming low-FODMAPS fruits and vegetables could be associated with benefits in intestinal health, especially in those who suffer from intestinal symptoms [44]. Additionally, a systematic review and meta-analysis found sufficient inconsistencies to preclude recognition that consuming low-FODMAPS fruits and vegetables can reduce gastrointestinal symptoms [45]. Additionally, Baker et al., in an old and widely cited study carried out with adults, reveals, as in this work, that men often had lower consumption of fruits and vegetables compared to women [46]. Not eating vegetables could be a factor that increased the risk of functional intestinal disorders. We found that men were more likely to avoid consuming vegetables and fruits in their daily diet than women (95% CI 1.31, 5.20). In contrast, Guo et al., in their case-control study, noted that people with irritable bowel syndrome ate more vegetables and legumes than controls [47].

#### 4.2.3. Beverages and Water Intake

In our study, the type of beverages that the participants drank was not investigated; however, there are studies that seem to reveal that the intake of soft drinks is associated with an increased risk of suffering from inflammatory bowel conditions, while, in contrast, the intake of tea can reduce it [37]. In a study similar to ours, IBS was found to be significantly associated with reduced water intake (as well as higher prevalence among women and low consumption of fruits and vegetables) [48]. We observed that drinking less than recommended significantly increased the risk of a functional intestinal condition; in addition, we found significant differences in water consumption between the sexes. In contrast, Salari-Moghaddam et al. (2020) reported a double risk of having irritable bowel syndrome among those who drank eight glasses or more water/day compared with those who drank two glasses or less. On the other hand, our casuistry failed to see an association between water consumption and IBS diagnosis; it is also important to note that these authors found no difference between water intake between women and men [49]. Furthermore, in the work by Zaribaf et al., it was found that those who drink many fluids during, before, or after meals have a lower risk of developing a functional bowel disorder [50].

### 4.3. Body Mass Index

Various reports agree that there are significant associations between people with obesity and a higher prevalence of functional bowel disease and gastrointestinal symptoms in general [51,52,53].

In our study, the higher the BMI, the greater the intestinal symptoms, with higher abdominal pain, distention, and diarrhea standing out. In addition, the risk for intestinal health increased proportionally and significantly to the participants’ body weight. People with overweight and obesity present factors that increase intestinal symptoms. Mechanisms linking obesity and gastrointestinal symptoms could be low-grade inflammation and adipocytes-released peptides. Obesity could affect gastrointestinal motility for the increased abdominal pressure. The release of pro-tumoral factors and pro-inflammatory cytokines from visceral fat is associated with low gastrointestinal motility [6]. However, in the literature, the association between obesity as a risk factor for functional gastrointestinal disorders seems to be less clear [54,55,56].

This work found a 70% prevalence of overweight and obesity among the participants. The prevalence of overweight and obesity has increased globally, affecting two out of three adults. In 2016, 12.3% of all deaths in the world were attributed to being overweight. The 2021 National Health and Nutrition Survey reports that the prevalence of overweight and obesity (BMI ≥ 25 kg/m^2^) in the Mexican population was 75.0% in women and 69.6% in men. The combined prevalence of overweight plus obesity (BMI ≥ 25 kg/m^2^) in 8 years increased by 0.2% in men, and 2.7% in women [57].

In the same way. The prevalence of reports of functional intestinal disorders, and intestinal disease in general, now seem to be higher worldwide and affect women more frequently [19].

### 4.4. Physical Activity

The weekly frequency of aerobic exercise was assessed among our participants. The trend directly proportional to the GIH test score did not surprise us. Physical activity-induced reduction in adiposity has shown benefits in improving obesity-mediated inflammation and oxidative stress status [58]. In this regard, there are recent and very interesting contributions that demonstrate the benefit of exercise on the intestinal microbiota and its effect on intestinal metabolism [59].

In addition, physical training has been confirmed to reduce intestinal inflammation and regulate gut microbiota profiles in people with insulin resistance [60].

Insulin resistance, as is known, is prevailing in people overweight or obese [61].

### 4.5. Food Schedule

We wished to recognize whether changes in feeding schedules affected gastrointestinal health, on the basis that the disruption of the circadian cycle can result in various gastrointestinal diseases [62]. However, we found no association of bowel symptomatology with meal times, where participants eat, or specific food groups. These unknowns could possibly be resolved with a larger sample size, in the general population, and with a Likert-type questionnaire indicating specific times and places.

### 4.6. Educational Level and Sociodemographic Characteristics

In this work, there was a significantly high risk in certain variables, such as age, gender, marital status, and occupation, on GIH. In contrast, Latif et al. found no association of these independent variables in their work [63].

The average of our sample had a high educational level, with more than 60% having completed university studies. A study based on the population of South Korea indicates that in rural populations a high level of education increases the probability of being obese by 19.7% [64].

However, Ogden et al. [65] reported that the prevalence of obesity in the US, adjusted for age, was lower in the high-income group and the prevalence of obesity among college graduates was lower (27.8%) than among those with some college education (40.6%) and those who were high school graduates or below (40.0%). Several socioeconomic sectors have a high prevalence of obesity [19,57] and this condition has been consistently associated with intestinal disorders [64,65], as can be observed in our sample.

Finally, this association of educational level, obesity, and intestinal disorders was studied by Mansouri et al. [66], who found—as we did in this article—that the main contributors to suffering from IBS were educational level, age, and marital status. We must insist that all factors must be considered, but mainly the control of eating habits and a sedentary lifestyle, to avoid the development of overweight and, jointly, intestinal disorders. It should be reiterated that the problem has not diminished despite knowledge of this issue; hence, the pertinence of our study.

### 4.7. Limitations and Strengths

In this study, interviewees’ memory was utilized to refer to various topics from the past, at least from the last month of their life, so we do not exclude the possibility of a memory bias.

On the other hand, the consumption of drugs that could improve gastrointestinal symptoms was questioned. However, the consumption of drugs that could give rise to the symptoms was not evaluated, such as the consumption of NSAIDs, steroids, and antimicrobials, among others.

Food consumption investigated was by food groups without specifications; the generality is reported here.

We used the Rome IV criteria. However, interesting previous publications have found differences in the prevalence of irritable bowel syndrome based on the use of the Rome III criteria compared to those of Rome IV [19]; therefore, the consensus on the diagnostic sensitivity between both is still controversial.

One of our strengths was the characteristics sample; they allowed the verification of vulnerability to GIH in a group of adults without non-transmissible chronic diseases and who were productive economically, especially women. At this age, people can develop diabetes or hypertension, which increases the severity of functional intestinal disorders. Our study offers information that can support future research with different levels of knowledge since we show evidence identifying early GI symptoms with a GIH test before the development of any chronic GI disease. We show the importance of maintaining a healthy body weight, diet, and lifestyle. Moreover, we verified that maintaining an adequate BMI could lead to fewer intestinal symptoms. Our research highlighted that the consumption of drugs to decrease symptoms did not help, as the people believed; in contrast, it was associated with more symptoms. Therefore their use should be re-evaluated according to the risk of GIH shown in our results.

## 5. Conclusions

These results could explain the prognostic value of eating habits and the importance of paying attention to body weight to reduce the risk of gastrointestinal diseases. On the other hand, it also questions the limited effect of the medication used to relieve gastrointestinal symptoms and its possible paradoxical results.

## Figures and Tables

**Table 1:** General sociodemographic and clinical characteristics of participants surveyed on intestinal symptoms (n = 250).

	Median	Percentile (25, 75)	Range (min, max)
Age (years)	38.5	25, 47	18, 63
Weight (kg)	70.5	61.0, 79.2	42, 120
Height (cm)	160	155.0, 165.2	142, 184
BMI (kg/m^2^)	27	24, 30	18, 50
		**Frequency (%)**	
Body Mass Index			
	Underweight	1	0.4
	Normal weight	75	30
	Overweight	106	42.4
	Obesity	68	27.2
Sociodemographic Data			
Marital status			
	Single	131	52.4
	With couple	119	47.6
Occupation			
	Paid	104	41.6
	Unpaid	146	58.4
Scholarship			
	Elementary	4	1.6
	Middle school	11	4.4
	High school	68	27.2
	Bachelor	158	63.2
	Other	9	3.6
Socioeconomic level			
	High	7	2.8
	Medium	234	93.6
	Low	9	3.6

**Table 2:** Frequency of intestinal symptoms at least once a week n(%).

Symptom	Total	Women (n = 199)	Men (n = 51)	*p*-Value
Diarrhea	40 (16)	36 (18.1)	4 (7.8)	0.75
Constipation	77 (30.8)	68 (34.2)	9 (17.6)	0.02
Flatulence	151 (60.4)	118 (59.3)	33 (64.7)	0.48
Abdominal distension	162 (64.8)	130 (65.3)	32 (62.7)	0.73
Abdominal heaviness	87 (34.8)	73 (36.7)	14 (27.5)	0.21
Abdominal pain	78 (31.2)	66 (33.2)	12 (23.5)	0.18

**Table 3:** Risk factors for gastrointestinal health. General Linear Model. (n = 250).

Exposure Variable		Mean Differences	CI 95%	ETA^2^	*p* Value
Water consumption per day * (reference group: less than one liter)	Between one and two liters Between two and three liters	−2.15 −2.91	−3.90, −0.41 −4.71, −1.10	0.046	0.010 <0.001
Consumption of 2 or more chocolate/week	Yes vs. No	0.06	−1.29, 1.41	0.001	0.931
Consumption of 2 or more cupcakes/week	Yes vs. No	−6.77	−2.00, 0.67	0.005	0.324
Alcoholic beverages intake, at least once a week	Yes vs. No	1.21	0.05, 2.37	0.017	0.041
Smoking, more than two times a week	Yes vs. No	−0.32	−1.48, 0.83	0.001	0.584
Irritating or spicy foods, at least once a week	Yes vs. No	0.99	−0.97, 2.96	0.005	0.319
Carbonated Drinks, at least once a week	Yes vs. No	−0.61	−1.97, 0.73	0.004	0.368
Coffe at least once a day	Yes vs. No	0.70	−0.70, 2.12	0.005	0.325
Eat Out	Yes vs. No	0.93	−0.49, 3.18	0.009	0.149
A usual morning snack	Yes vs. No	−0.513	−2.46, 1.43	0.001	0.605
A usual evening snack	Yes vs. No	−1.93	−4.07, 0.20	0.014	0.076
Consumption vegetables * (reference group: less than once a week)	One time a week Every 3 or 4 days Every 1 or 2 days Daily	−1.96 −3.57 −5.09 −6.02	−4.35, 0.44 −5.74, −1.39 −7.50, −2.67 −8.23, −3.81	0.268	0.215 <0.001 <0.001 <0.001
Weekly exercise	Once Every 3 or 4 days Every 1 or 2 days Daily	−1.35 −3.00 −4.71 −6.60	−2−28, −0.42 −4.68, −2.51 −5.75, −3.67 −7.70, −5.50	0.400	0.001 <0.001 <0.001 <0.001
Body mass index (kg/m^2^) * (reference group: 24.9 or less)	40 or more 35 to 39.9 30 to 34.9 25 to 29.9	−5.40 −4.47 −2.58 −1.12	−9.37, −1.44 −6.87, −2.08 −4.33, −0.84 −2.53, 0.30	0.169	0.002 <0.001 <0.001 0.203
Symptomatic medication use * (reference group: no medication)	Daily Every 3–4 days Every 1–2 days 1 per week	−4.70 −4.13 −5.74 −2.46	−6.09, −3.30 −5.64, −2.62 −7.61, −3.87 3.31, −1.62	0.279	<0.001 <0.001 <0.001 <0.001

* Post Hoc Test: Bonferroni.

**Table 4:** Distribution of Irritable Bowel Syndrome (IBS), according to the Rome IV Criteria.

	With Functional Bowel Disorder		Without Functional Bowel Disorder	
	n	%	n	%
With constipation	65	56.0	52	38.8
With diarrhea	4	3.40	9	6.7
Mixed	15	13.0	10	7.4
Indeterminate	32	27.6	0	0
Asymptomatic	0	0	63	47.0
Total	116	100	134	100

## Data Availability

The data presented in this study are available from the corresponding author upon reasonable request.

## Abbreviations

GI	Gastrointestinal
GIH	Gastrointestinal health
FGID	Functional gastrointestinal disorders
IBS	Irritable bowel syndrome
BMI	Body mass index
WGO	World Gastroenterology Organization
LRA	Logistic regression analysis
FODMAP	Fermentable oligosaccharides, disaccharides, monosaccharides, and polyols
